# Immunosuppressive therapies in scorpion envenomation: new perspectives for treatment

**DOI:** 10.3389/ftox.2024.1503055

**Published:** 2024-11-19

**Authors:** Mouzarllem Barros Reis, Eliane Candiani Arantes

**Affiliations:** Departamento de Ciências BioMoleculares, Faculdade de Ciências Farmacêuticas de Ribeirão Preto, Universidade de São Paulo, Ribeirão Preto, Sao Paulo, Brazil

**Keywords:** corticosteroids, scorpion envenoming, immune response, scorpionism, imunossupression

## Abstract

Scorpion envenoming is a relevant and neglected public health problem in some countries. The use of antivenom is widespread in many regions, targeting specific species of scorpions. However, the uncontrolled proliferation and adaptation of these animals to urban environments, combined with limited access to treatments in remote areas and delays in antivenom administration contribute to a significant number of fatalities from scorpion-related incidents. In recent decades, new research has revealed that the immune system plays an important role in triggering immunopathological reactions during scorpion envenoming, which places it as a therapeutic target; however, few clinical studies have been conducted. This work provides a review of the main immunopathological aspects of scorpion envenoming, as well as the clinical trials conducted to date on the use of corticosteroids for the treatment of scorpionism. We highlight emerging treatment perspectives as well as the need for further clinical trials. The use of corticosteroids in scorpionism, when appropriate, could significantly enhance access to treatment and help reduce fatalities associated with scorpion stings.

## 1 Introduction

Scorpionism is still a major public health problem in countries where species of medical importance are endemic (Chippaux and Goyffon, 2008). Scorpion stings are responsible for severe clinical symptoms, and venoms have a wide range of effects on various biological systems (Isbister and Saluba Bawaskar, 2014). Scorpion venoms contain many bioactive compounds, including hyaluronidases (which act as a venom spreading factor), metalloproteinases, serine proteases, and ion channel modulating toxins, which are responsible for the neurotoxic manifestations of scorpionism and activation of the immune system during envenoming (Pucca et al., 2015).

Although several classifications of severity have been postulated over the years, the most accepted one classifies clinical outcomes into three distinct groups: local, moderate and severe manifestations (Isbister and Saluba Bawaskar, 2014). Local manifestations generally include intense pain (often requiring anesthetic block), hyperemia, paresthesia and loss of function of the affected limb (Reis et al., 2019). It is important to emphasize that most scorpion stings cause only local symptoms (Cupo, 2015). Moderate manifestations include mild clinical repercussions such as vomiting, sweating, priapism, gastrointestinal disorders, miosis, bradycardia, hypotension (parasympathetic manifestations), as well as tachycardia, mydriasis, hypertension, convulsions, hyperglycemia (sympathetic manifestations). Severe manifestations include two major events responsible for death: pulmonary edema and cardiogenic shock (Isbister and Saluba Bawaskar, 2014).

The venom of several scorpion species can induce a robust activation of the immune response, and these processes have important clinical and immunopathological repercussions in scorpion envenoming (Reis et al., 2019). Classically, toxins are recognized by pattern recognition receptors (PRRs) of innate immunity such as Toll-like receptor 2, 4 and CD14, which initiate an intracellular signaling cascade culminating in the activation of the pro-inflammatory transcription factor NF-κB, inducing the production of pro-inflammatory mediators (Zoccal et al., 2014).

After venom inoculation, systemic immunological repercussions are observed in both murine models and human (Reis et al., 2019). There is a robust increase in the number of leukocytes, which recognize venoms through venom-associated molecular patterns (VAMPs) (Zoccal et al., 2014), leading to a systemic hyperactivation of an immune response, a sepsis-like event, which has a direct role in the dysfunctions observed in cases of severe envenoming. In addition, pulmonary inflammation induced by scorpion toxins is pivotal for mortality induced in envenoming cases, as well as cardiac, renal, hepatic, pancreatic dysfunctions, among others (Wiezel et al., 2024).

Over the years, the production and technology of heterologous antivenoms has been improved. However, as an immunobiological, it has limitations regarding distribution in all locations, being generally centralized in large urban centers (Ozkan et al., 2005). The low availability of antivenom in remote regions is a factor responsible for most cases of death, mainly in children and the elderly (Martins et al., 2024). Another important facet of antivenom is that the time to its administration is a concern, as explored by researchers who showed that delayed administration of antivenom allowed immune response activation induced by venom and contributed directly with cardiac manifestations and mortality during envenoming in a canine model (Tarasiuk et al., 1998). Importantly, the role of the immune system has been shown to be an important pathophysiological mechanism of scorpionism, sometimes overriding the neurotoxic mechanisms of venoms (Reis et al., 2019).

The present review aims to promote a more critical look at the importance of clinical studies that include the treatment of scorpionism with corticosteroids, thus covering all areas of immunopathological mechanisms that contribute to the morbidity and mortality of this neglected event.

## 2 Considerations on the epidemiology of scorpionism

Scorpion stings have always been considered a public health problem in several countries around the world. Some specific regions, such as South Africa (Marks et al., 2019), Mexico (Hernández-Muñoz et al., 2024), the Middle East (Amr et al., 2021) and South America (especially Brazil) (Guerra-Duarte et al., 2023), are places with high incidence and prevalence of these accidents. Scorpion stings are well managed due to access to tertiary health centers and the availability of antivenom for the species that causes the accidents. However, the number of deaths from scorpion stings remains high, despite the seemingly well-established clinical protocols and the development of highly effective heterologous antivenoms (Guerra-Duarte et al., 2023; Martins et al., 2021).

The last relevant study on the global epidemiology of scorpionism was published in 2008 (Chippaux and Goyffon, 2008). Despite the scarcity of updated epidemiological data and the underreporting of scorpion accidents in several countries, even the underestimated numbers are cause for concern. In 2022, Brazil recorded an incredible number of 183,738 cases of accidents, with 92 deaths recorded. This number makes the scorpionism scenario alarming, since Brazil is one of the world’s main producers of heterologous antivenom (Guerra-Duarte et al., 2023).

## 3 Immune-target therapies in murine models

Studies conducted in mouse models are one of the main methods applied to investigate the pathophysiology of scorpion envenoming. Although clinical studies in humans have described some of the main pathophysiological mechanisms (Cupo, 2015), access to murine models has allowed advances in the understanding of the changes that occur during envenoming, specifically the role of immune system during moderate and severe cases (Reis et al., 2019).

In 1963, Greenberg and Ingalls first reported the impact of hydrocortisone during *Androctonus autralis hector* (Aah) envenoming, in which 15 compounds were tested to evaluate the reduction in mortality in mice, among them, hydrocortisone at a dose of 25 mg/kg, with no impact on mortality compared to the control (sterile saline solution) (Greenberg and Ingalls, 1963). For the same species, tacrolimus (a macrolide from *Streptomyces tsukubaensis* with immunosuppressive properties) were inoculated every 12 h for 21 days (1 mg/kg) before envenoming with sublethal dose of Aah venom (10 μg/20 g of mice), decreasing several parameters analyzed such as hyperglycemia and pulmonary edema. When mice were exposed to the same protocol with tacrolimus and challenged with lethal dose of Aah venom (72.41 μg/20 g of mice), animals had increased survival time (Kabrine and Laraba-Djebari, 2014).

Regarding other species, *Leiurus quinquestriatus* has only one study in the literature relating the use of anti-inflammatory drugs and clinical outcome. In this study, the administration of montelukast (a leukotriene receptor antagonist – 10 or 20 mg/kg, orally), hydrocortisone (corticosteroid – 5 or 10 mg/kg, intravenously) and indomethacin (cyclooxygenase inhibitor – 10 or 20 mg/kg, intravenously) were administered beforehand, and then the mice were challenged with doses of 0.25 or 0.30 mg/kg of venom. All three drugs analyzed were able to increase the survival of the animals in a 24-h observation period, with montelukast being more effective (Abdoon et al., 2005).

Most of the research in the scientific literature on the impact of anti-inflammatory and immunosuppressive therapies in the treatment of scorpionism are focused on the venom of the Brazilian scorpion *Tityus serrulatus* (yellow scorpion) (Reis et al., 2019). A research conducted by Zoccal and collaborators demonstrated that the treatment of mice envenomated with the toxins Ts2 and Ts6 and treated with MK-886 (a 5-lipoxygenase inhibitor – 5 mg/kg, orally) and celecoxib (a selective COX-2 inhibitor – 5 mg/kg, orally) resulted in a decrease in the inflammatory infiltrate in the peritoneal cavity (the site of inoculation of the toxins) (Zoccal et al., 2013). In another large study by the same group, the challenge of animals with a lethal dose of *Tityus serrulatus* venom (TsV) and treatment with indomethacin (2 mg/kg, intraperitoneally) was able to reduce the mortality of the challenged mice. This study demonstrated that the balance of prostaglandins and leukotrienes is a pivotal mechanism that control interleukin-1β (IL-1β) production, a main mediator involved in the pathophysiology of pulmonary edema (a manifestation of severe envenoming cases) (Zoccal et al., 2016).

IL-1β has been demonstrated in several studies with TsV as a key inflammatory mediator in the pathophysiology process in severe cases during envenoming. In another study by Zoccal et al., the production of IL-1β was investigated focusing on the innate immune receptor cluster of differentiation 14 (CD14) and the scavenger receptor CD36. While CD14 induces the production of prostaglandin E_2_ (PGE_2_), with consequent increase of IL-1β, CD36 induces the production of leukotriene B4 (LTB_4_) and decrease in IL-1β production. This mechanism was corroborated using U-75302 (LTB_4_ receptor antagonist – 50 ng/mouse, intranasally) to treat animals inoculated with the lethal dose of TsV (180 μg/kg), which had increased mortality, as well as worse pulmonary edema outcomes (Zoccal et al., 2018). To corroborate the hypothesis of CD36 in promoting a reduction in fatal outcomes in TsV envenoming, the authors demonstrated in another study that the use of EP80137 (an agonist of the scavenger receptor CD36–0.289 μmol/kg, intraperitoneally) promoted an increase in the production of LTB_4_, a decrease in IL-1β, and a reduction in mortality and pulmonary edema in a murine envenoming model challenged with a lethal dose of TsV (Zoccal et al., 2019).

The immune system appears to be involved in the pathophysiology of other clinical manifestations during scorpion envenoming, such as hyperglycemia (which is a predictor of clinical severity in humans) and cardiac alterations that lead to cardiogenic shock (Cupo, 2015; Miranda et al., 2015). Reis et al. demonstrated that transient hyperglycemia in cases of TsV envenoming is due to an increase in IL-1β production in the pancreas, which via the IL1R receptor induces an increase in nitric oxide (NO), causing disturbance in insulin release, leading to hyperglycemia. Mice treated with the nitric oxide synthase inhibitor N(G)-Nitro-L-arginine methyl ester (L-NAME – 100 mg/kg, intraperitoneally) did not present hyperglycemia (Reis et al., 2020a). A large study by the same group demonstrated that cardiac alterations observed in severe TsV envenoming are caused by excessive release of IL-1β and PGE_2_, which synergically with neurotoxins increase the release of acetylcholine (ACh) leading to cardiogenic shock. In this study, animals treated with dexamethasone at an immunosuppressive single dose (5 mg/kg, intraperitoneally) were able to prevent cardiac alterations and mortality. The use of dexamethasone was shown to be effective in a time-dependent manner, indicating the use of this corticosteroid to prevent mortality from scorpion envenoming until patients have access to antivenom (Reis et al., 2020b). The use of dexamethasone (5 mg/kg, intraperitoneally) was also able to prevent mitochondrial alterations in cardiomyocytes (Reis et al., 2023) and pulmonary edema (Yamashita et al., 2020) in other studies. The mains outcomes of immune-target drugs in murine models during scorpion envenoming are highlighted in Table 1.

**TABLE 1:** Target immune system therapies in murine models.

Species	Drug	Dose	Mechanism of action	Outcome	Reference
*Androctonus australis hector*	Hydrocortisone	25 mg/kg	Immunosuppressant	No difference in survival time	Greenberg and Ingalls (1963)
	Tacrolimus	1 mg/kg	Immunosuppressive effect by inhibiting calcineurin	Decreased pulmonary edema, exudation, hyperglycemia, protection against mortality	Kabrine and laraba-Djebari (2014)
*Leiurus quinquestriatus*	Montelukast Hydrocortisone Indomethacin	10–20 mg/kg 5–10 mg/kg 10–20 mg/kg	LTB4 receptor antagonist Immunosuppressant Nonselective COX inhibitor	Prolonged survival time, with better results for montelukast	Abdoon et al. (2005)
*Tityus serrulatus*	MK-886 Celecoxib	5 mg/kg 5 mg/kg	5-lipoxygenase inhibitor COX-2 inhibitor	Deceased leukocyte influx to the site of inoculation	Zoccal et al. (2013)
	Indomethacin	2 mg/kg	Nonselective COX inhibitor	Prolonged survival time and decreased lung inflammation	Zoccal et al. (2016)
	U75302	50 ng	LTB4 receptor antagonist	Decreased survival time and increased lung inflammation	Zoccal et al. (2018)
	EP80317	0.289 μmol/kg	CD36 selective ligand	Prolonged survival time and decreased lung inflammation	Zoccal et al. (2019)
	L-NAME	100 mg/kg	Nitric oxide synthase inhibitor	Decreased pancreas inflammation and reduced hyperglycemia	Reis et al. (2020a)
	Dexamethasone	5 mg/kg	Immunosuppressant	Prolonged survival and improved cardiac function	Reis et al. (2020b)
	Dexamethasone	2 mg/kg	Immunosuppressant	Reduced inflammation and edema in the lungs	Yamashita et al. (2020)
	Dexamethasone	5 mg/kg	Immunosuppressant	Preserved cardiac mitochondrial function	Reis et al. (2023)

## 4 Use of corticosteroids in human-conducted studies

Studies using corticosteroids to treat scorpion stings in humans are scarce in the literature and present problems regarding the dose and potency of the drugs chosen. In the literature, only three studies with scorpions from Tunisia (without specifying the species that caused the accidents) using hydrocortisone as a therapeutic approach for analyzing clinical outcomes and mortality are described. In a study conducted by Abroug et al., 600 patients over 10 years old victims of scorpion stings were randomly assigned to receive hydrocortisone hemisuccinate at a dose of 50 mg/kg or placebo. No significant differences were found in the length of hospital stay and mortality during the 4-h clinical evaluation period (Abroug et al., 1997). In another study by Bahloul et al., a case-control study was conducted with 184 children admitted due to scorpion envenoming between 1990 and 2002, demonstrating that there was no difference in mortality or length of stay in the intensive care unit (ICU) in the groups with hydrocortisone and steroid-free patients (Bahloul et al., 2013). Another case-control study, also by the same group, analyzed 48 patients over 16 years old showed no statistical difference in the use of mechanical ventilation, ICU stay length and ICU mortality in groups that received or not hydrocortisone (Bahloul et al., 2014). In all studies, the rationale for the use of hydrocortisone was that this drug was part of the protocol for all scorpion-envenomed patients at some hospitals in Tunisia, with no details on studies that indicated this protocol. The main outcomes on human-conducted studies are highlighted in Table 2.

**TABLE 2:** Corticoid therapy for scorpion envenoming conducted in humans.

Species	Drug	Outcome	Number of patients	Reference
Scorpion accidents from Tunisia (species not informed)	Hydrocortisone (50 mg/kg, 6/6 h - max. 3 g)	No difference in mortality or hospital stay	600 patients over 10 years old	Abroug et al. (1997)
	Hydrocortisone (Case-control study)	No difference in mortality or Intensive Care Unit stay	184 children under 16 years old	Bahloul et al. (2013)
	Hydrocortisone (Case-control study)	No difference for use of medical ventilation, mortality or Intensive Care Unit stay	48 patients over 16 years old	Bahloul et al. (2014)

## 5 Discussion

The pathophysiology of scorpion envenoming has, over the last few years, pointed to a robust role of the immune system in severe manifestations of scorpionism (Reis et al., 2019). In a 1998 study by Tarasiuk et al., in which the authors used a model of envenoming by *L. quinquestriatus* in dogs, it was observed that while renal clearance of the venom occurs rapidly, the symptoms of acute pulmonary edema and cardiogenic shock persist for a longer period of time, due to factors activated by the venom and not by the action of the venom itself, rendering antivenom ineffective in cases where delayed administration occur (Tarasiuk et al., 1998). Of importance, there are studies in literature that point to an increase in circulatory cytokines in victims of scorpionism, such as IL-1β, IL-6, IL-8, IL-10 and TNF-α (Fukuhara et al., 2003).

With increasing evidence of immunological involvement in scorpionism, the use of therapies targeting the immune system, especially corticosteroids, has shown robust results in murine models (Reis et al., 2020b). Studies with the venom of the scorpions *T. serrulatus* and *L. quinquestriatus* have shown a reduction in mortality, especially by attenuating the two most relevant manifestations of scorpion envenoming: pulmonary edema and cardiogenic shock (Abdoon et al., 2005; Reis et al., 2020b; Zoccal et al., 2016). Although low doses of corticosteroids are used in the treatment of *L. quinquestriatus*, mortality in mice inoculated with TsV requires high doses of corticosteroids in a single application after envenoming (Reis et al., 2020b). Research with humans that aimed to evaluate the role of corticosteroid use in clinical outcomes have mostly used low doses of low-potency corticosteroids (Abroug et al., 1997; Bahloul et al., 2014; Bahloul et al., 2013).

Due to the small number of clinical trials, the conduction of studies about the use of corticosteroids for scorpion envenoming deserves special attention and new reformulations. First, the use of high-potency corticosteroids in high doses may be a crucial factor for the correct assessment of clinical improvement. Human studies use hydrocortisone in clinical trials, and this corticosteroid is also routinely used in some services prior to the administration of heterologous antivenom (Abroug et al., 1997; Bahloul et al., 2014; Bahloul et al., 2013). Hydrocortisone is pharmacologically a low-potency corticosteroid, corresponding to endogenous cortisol, with an average duration of clinical effects ranging from 8 to 12 h (William, 2018). On the other hand, the study with a murine model that showed the effectiveness of corticosteroids in the treatment of scorpionism used dexamethasone (5 mg/kg, single dose), a drug 20 times more potent than hydrocortisone, and biological action of 36 h (3 times longer) (Reis et al., 2020b). The immunosuppressive dose demonstrated by Reis et al. may be the answer for new clinical studies that use potent corticosteroids in high doses (Reis et al., 2020b). Importantly, Patrão-Neto and collaborators demonstrated that the use of dexamethasone also has an impact on the clinical course of *Bothrops* accidents at a dose of 1 mg/kg, reducing myotoxicity and activation of immune cells after envenoming (Patrão-Neto et al., 2013).

Also, the administration time needs to be assessed in protocols in which injured patients have rapid access to corticosteroid until they can get access to the antivenom. It is extremely important to emphasize that the use of corticosteroids is not intended to replace antivenom therapy, but rather to complement it, since studies show that the earlier the immune response is inhibited, the better the clinical outcome in a murine model. Another important factor is that neurotoxins need to be neutralized quickly, making the use of corticosteroids prior to antivenom a treatment proposal that covers both pathological arms of this condition: immune response and ion channel-modulating toxins (Reis et al., 2020b).

There are several clinical protocols for the use of immunosuppressants for numerous diseases, with their respective doses (Timmermans et al., 2019). A look at these protocols can help in the design of clinical studies that aim to evaluate the role of immunosuppression in the context of scorpion envenoming. For example, the use of dexamethasone in pediatrics has a well-established maximum safe dose in the literature of 0.6 mg/kg in cases of viral croup (Cruz et a., 1995). The use of tacrolimus in autoimmune conditions is also classically safe at a dose of 1.5 mg/kg in chronic use for more than 24 weeks (Yoo et al., 2021). Other human pathologies that use immunosuppressive drugs are excellent guides for choosing the dose in clinical trials for this type of investigation.

Mortality from scorpion envenoming is still a relevant and neglected public health event. Since the emergence of antivenom, it has been assumed that neutralizing the venom is the only relevant factor for clinical treatment, a hypothesis that is not corroborated by the various fatal outcomes that occur even after administration of the antivenom. The translation of the hypotheses obtained in mice can be of great value so that deaths due to scorpionism (mainly in children) become a purely theoretical framework.
